# Risk factors analysis of hyperbilirubinemia after off-pump coronary artery bypass grafting: a retrospective observational study

**DOI:** 10.1186/s13019-021-01678-6

**Published:** 2021-10-10

**Authors:** Yingdi Gao, Dongjie Li, Honghong Dong, Yulin Guo, Yuanshu Peng, Yan Liu, Pixiong Su

**Affiliations:** 1 Department of Cardiac Surgery, Heart Center and Beijing Key Laboratory of Hypertension, Beijing Chaoyang Hospital, Capital Medical University, No. 8 Gong Ti South Road, Chaoyang District, Beijing, 100020 China; 2 Department of Thoracic Surgery, Beijing Chaoyang Hospital, Capital Medical University, Beijing, 100020 China

**Keywords:** Off-pump coronary artery bypass grafting, Hyperbilirubinemia, Risk factors

## Abstract

**Background:**

Hyperbilirubinemia is a common complication after off-pump coronary artery bypass grafting (OPCAB), but the incidence and the risk factors are unclear. This study aimed to analyze the incidence and risk factors of postoperative hyperbilirubinemia in patients undergoing OPCAB.

**Methods:**

From December 2016 to March 2019, a total of 416 consecutive patients undergoing OPCAB were enrolled in this single-center retrospective study. Patients were divided into the normal serum total bilirubin group and the hyperbilirubinemia group based on the serum total bilirubin levels. Perioperative variables between the two groups were compared by univariate logistic regression analysis. Then, multivariate binary logistic regression analysis was used to analyze the independent risk factors of developing hyperbilirubinemia in patients underwent OPCAB. *P* < 0.05 was considered as statistically significant.

**Results:**

Thirty two of 416 (7.7%) patients developed postoperative hyperbilirubinemia. Univariate regression analysis showed significant differences in gender (73.96% vs. 93.75%, *P* = 0.012), preoperative total bilirubin levels (11.92 ± 4.52 vs. 18.28 ± 7.57, *P* < 0.001), perioperative IABP implantation (22.66% vs. 43.75%, *P* = 0.008), perioperative blood transfusion (37.50% vs. 56.25%, *P* = 0.037) between the two groups. Multivariate logistic regression analysis revealed that elevated preoperative serum total bilirubin levels (OR = 1.225, 95% *CI* 1.145–1.310, *P* < 0.001), perioperative blood transfusion (OR = 4.488, 95% *CI* 1.876–10.737, *P* = 0.001) and perioperative IABP implantation (OR = 4.016, 95% *CI* 1.709–9.439, *P* = 0.001) were independent risk factors for hyperbilirubinemia after OPCAB.

**Conclusions:**

Hyperbilirubinemia is also a common complication after OPCAB. Elevated preoperative serum total bilirubin levels, perioperative blood transfusion, and perioperative IABP implantation were independent risk factors for patients developing hyperbilirubinemia after OPCAB. Further studies need to be conducted to confirm the risk factors of hyperbilirubinemia after OPCAB procedure.

## Background

Off-pump coronary artery bypass grafting (OPCAB) has been accepted by more and more cardiac surgeons due to its less neurological complications and lower in-hospital mortality [1]. Hyperbilirubinemia is a severe complication after OPCAB, and is associated with increased postoperative morbidity and mortality, ranging from 4.1 to 25%, due to the differences of demographic characteristics, surgery sites and techniques, cardiopulmonary bypass (CPB) time and cross-clamp time, sample size [2–9].

Whereas, the incidence of hyperbilirubinemia after OPCAB was unclear, since previous studies have not reached a consensus on the diagnostic criteria of perioperative hyperbilirubinemia, the incidence of hyperbilirubinemia after conventional CABG was significantly different, ranging from 13 to 51% [2–7, 9]. Meanwhile, few studies have elucidated the risk factors of hyperbilirubinemia after CABG. Mastoraki et al. and Sharma et al. have reported the hyperbilirubinemia after cardiac surgery, but they did not distinguish the surgical types, use of CPB and other factors [2, 3]. Nishi et al. and Chen et al. reported hyperbilirubinemia after valve and aortic surgeries, respectively [10, 11]. However, studies on the incidence and risk factor analysis of hyperbilirubinemia after OPCAB are still limited.

Thus, the present study aimed to detect the incidence and analyze the independent risk factors of postoperative hyperbilirubinemia after OPCAB.

## Materials and methods

### Study population

**Fig. 1:**
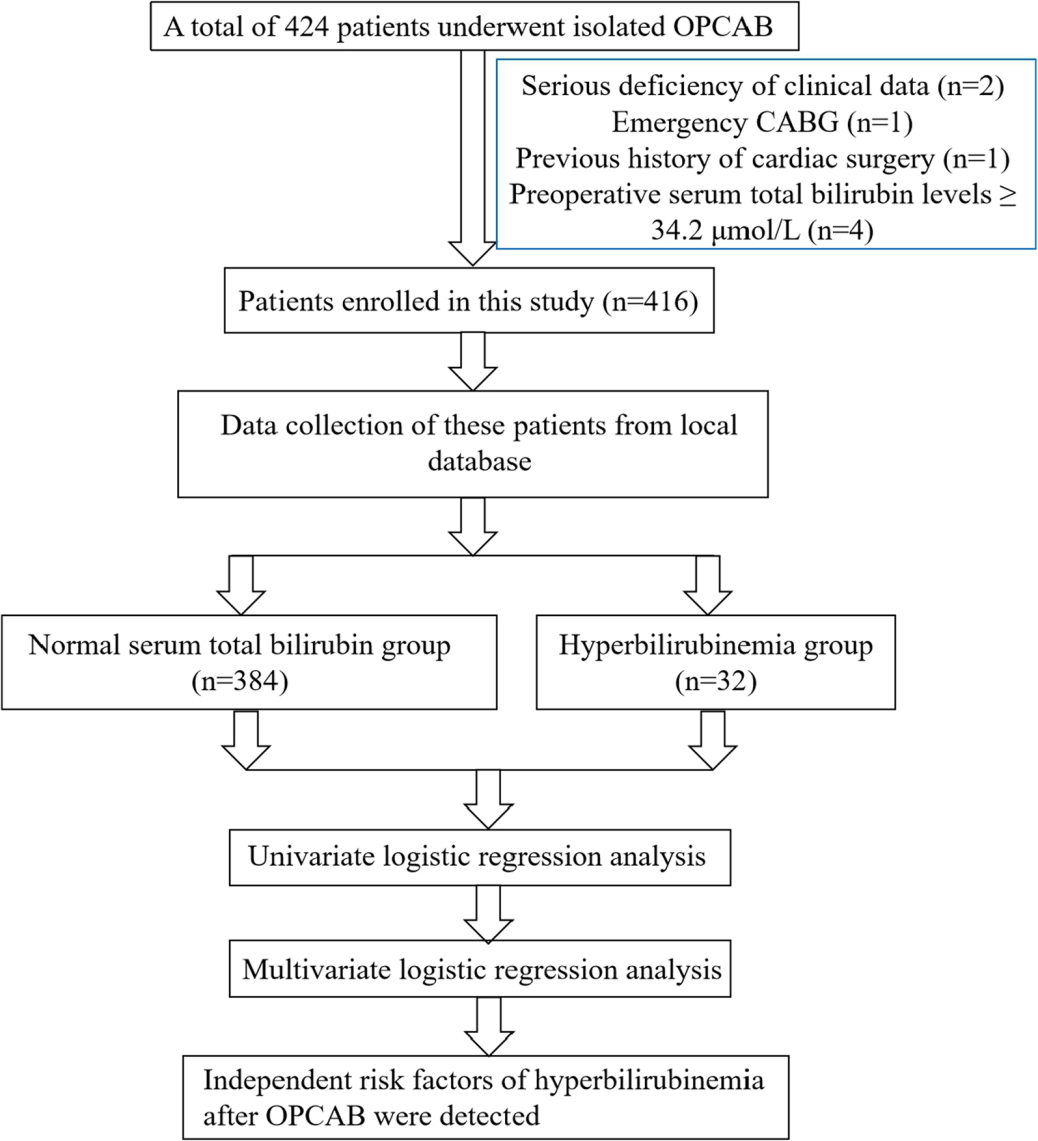
Flow chart of the study design. OPCAB, off-pump coronary artery bypass grafting

This was a single-center retrospective study conducted at Beijing Chaoyang Hospital. From December 2016 to March 2019, 424 consecutive patients undergoing isolated OPCAB were enrolled. The inclusion criteria were: 1. age ≥ 18; 2. patients undergoing isolated OPCAB. The exclusion criteria were: 1. serious deficiency of clinical data; 2. emergency CABG; 3. previous history of cardiac surgery; 4. preoperative serum total bilirubin levels ≥ 34.2 μmol/L. After exclusion, there were 416 patients enrolled in this study. Figure 1 shows the study flow.

The study was in accordance with the Declaration of Helsinki (as revised in 2013). It was authorized by local research ethics committee of Beijing Chaoyang Hospital, and subject informed consent for this retrospective study was waived.

### Data collections and definitions

Demographics variables, comorbidities, and perioperative data including age, gender, body mass index (BMI), smoking history, hypertension, diabetes, hyperlipidemia, chronic kidney disease (CKD), chronic obstructive pulmonary disease (COPD), peripheral vascular disease (PVD), cerebrovascular disease, prior myocardial infarction (MI), left ventricular end diastolic diameter (LVEDD), left ventricle ejection fraction (LVEF), biochemical indicators (albumin, liver enzyme, serum creatinine, urea nitrogen, etc.), target vessels, operation time, perioperative blood transfusion, and perioperative intra-aortic balloon pump (IABP) implantation were collected via local database of our center. Postoperative serum total bilirubin, direct bilirubin and indirect bilirubin levels were recorded on the first, third and fifth day after surgery routinely. For patients with high serum bilirubin level and continuous upward trend, measurement intervals were shortened and detection frequency were increased.

Key data elements were defined according to the “2020 AHA/ACC Key Data Elements and Definitions for Coronary Revascularization” [12]. Hyperbilirubinemia was defined as the occurrence of serum total bilirubin ≥ 34.2 μmol/L (twice the upper limit of normal value) at any time after surgery [13].

### Surgical procedures

Surgical procedures were performed by the same group of surgeons. Most patients were subjected to median sternotomy (n = 389, 93.5%), while some of the patients underwent minimally invasive direct coronary artery bypass (MIDCAB) or minimally invasive coronary artery bypass grafting (MICS-CABG), with a 6 cm incision in the fourth or fifth intercostal space (n = 27, 6.5%). The left internal mammary artery (LIMA) pedicle to left anterior descending artery (LAD) graft was used in all patients. Saphenous vein grafts (SVG) were harvested via endoscope (n = 378) or openly (n = 24).

### Statistical analysis

Statistical analyses were performed using SPSS 26.0 statistical software (IBM Corp. Released 2013. IBM SPSS Statistics for Windows, Version 26.0. Armonk, NY: IBM Corp). The Shapiro Wilk test was used to test the normality of measurement data. Measurement data of normal distributions were expressed as means ± standard deviation, and t-test was used for group comparisons. Measurement data of non-normal distributions were presented as *M* (*P*_25_, *P*_75_), and the non-parametric Mann–Whitney *U* test was used for group comparisons. Categorical variables were expressed as frequencies (percentage), and chi-square test or Fisher’s exact test were used for group comparisons. For variables that exhibited significant differences in univariate analysis, the multivariate binary logistic regression analysis model was used to obtain further conclusions. *P* < 0.05 was considered statistically significant.

## Results

### Baseline characteristics

A total of 416 patients undergoing OPCAB were enrolled, and the baseline characteristics were shown in Table 1. There were 32 patients developing hyperbilirubinemia after surgery, and the incidence of postoperative hyperbilirubinemia was 7.7%. The hyperbilirubinemia group had a higher basal serum total bilirubin level, a higher proportion of male patients, perioperative blood transfusion and perioperative IABP implantation compared with normal serum total bilirubin group. In the hyperbilirubinemia group, severe hyperbilirubinemia (serum total bilirubin ≥ 51.3 μmol/L) occurred in 6 patients (1.4%). In the hyperbilirubinemia group, the percentage of direct bilirubin to total bilirubin was 36.12% ± 6.32% before surgery, and 51.46% ± 19.98% after surgery, which was significantly different (*P*  < 0.001). There were 23 of hyperbilirubinemia patients (71.88%) had direct bilirubin levels elevation with a extent from 4.29% to 48.21%, and 4 patients died. There were 9 patients (28.13%) with indirect bilirubin levels elevation, and none of them died.

**Table 1:** Baseline characteristics of included patients

Variables^a^	All patients (n = 416)	Normal serum total bilirubin group (n = 384)	Hyperbilirubinemia group (n = 32)	*Z*/*t* /*χ*^2^	*P* value
Age ≥ 75 years	52(12.5)	49(12.8)	3(9.4)	0.310	0.783
Male	314(75.48)	284(73.96)	30(93.75)	6.252	0.012^**c**^
BMI, kg/m^2^	25.45 ± 3.21	25.45 ± 3.22	25.49 ± 3.04	0.073	0.942
Smoking history	217(52.16)	200(52.08)	17(53.13)	0.013	0.910
Comorbidities					
Diabetes	186(44.71)	175(45.57)	11(34.28)	1.498	0.221
Hypertension	289(69.47)	268(69.79)	21(65.63)	0.242	0.623
Hyperlipidemia	235(56.49)	215(55.99)	20(62.5)	0.509	0.475
CKD	4(0.96)	4(1.04)	0(0.00)	0.337	1.000
COPD	8(1.92)	7(1.82)	1(3.13)	0.266	0.476
Peripheral vascular disease	32(7.69)	30(7.81)	2(6.25)	0.102	1.000
Cerebrovascular disease	92(22.12)	82(21.35)	10(31.25)	1.679	0.195
Anemia	147(35.34)	139(36.20)	8(25.00)	1.621	0.203
NYHA III–IV	74(17.79)	66(17.19)	8(25.00)	1.233	0.267
Myocardial infarction occurring > 21 days before operation	191(45.91)	178(46.35)	13(40.63)	0.390	0.532
Left ventricular ejection fraction, %	60.71 ± 11.01	60.77 ± 10.93	60.03 ± 12.07	0.364	0.716
Left ventricular end diastolic diameter, cm	49.48 ± 5.81	49.42 ± 5.74	50.23 ± 6.70	0.762	0.446
Left atrial diameter, cm	37.44 ± 4.55	37.41 ± 4.55	37.78 ± 4.63	0.439	0.661
Pulmonary artery hypertension	8(1.92)	6(1.56)	2(6.25)	3.441	0.120
Absolute value of red blood cell, × 10^12^/L	4.31 ± 0.54	4.30 ± 0.54	4.45 ± 0.50	1.542	0.124
Albumin, g/L	39.72 ± 3.41	39.71 ± 3.38	39.87 ± 3.78	0.036	0.971
Serum creatinine, μmol/L^b^	73.70 (63.10, 87.25)	73.55 (62.60, 87.08)	76.75 (66.75, 88.30)	1.096	0.273
AST, U/L^b^	22.00 (17.00, 31.00)	22.00 (17.00, 30.00)	21.00 (17.25, 36.00)	0.036	0.971
ALT, U/L^b^	22.00 (16.00, 37.75)	22.00 (16.00, 30.00)	21.00 (14.25, 44.00)	0.361	0.718
ALP, U/L^b^	69.00 (57.00, 80.00)	68.50 (57.00, 79.00)	74.50 (56.25, 86.25)	1.342	0.180
GGT, U/L^b^	26.00 (18.00, 43.00)	26.00 (18.00, 41.00)	27.00 (19.25, 59.75)	1.513	0.130
Serum total bilirubin, μmol/L	12.41 ± 5.10	11.92 ± 4.52	18.28 ± 7.57	5.155	< 0.001^c^
Operation time, hours	3.89 ± 0.71	3.87 ± 0.71	4.01 ± 0.76	0.581	0.561
Intraoperative blood loss, ml	465.79 ± 176.90	463.33 ± 174.40	495.31 ± 205.32	0.522	0.601
Perioperative blood transfusion [14]	162(38.94)	144(37.50)	18(56.25)	4.367	0.037^c^
Perioperative IABP implantation	101(24.28)	87(22.66)	14(43.75)	7.149	0.008^c^
Target vessels^b^	3.00(2.00, 3.00)	3.00(2.00, 3.00)	3.00(2.00, 3.00)	0.343	0.732

*BMI* body mass index, *CKD* chronic kidney disease, *COPD* chronic obstructive pulmonary disease, *AST* aspartate aminotransferase, *ALT* alanine aminotransferase, *ALP* alkaline phosphatase, *GGT* γ-Glutamyl transpeptidase, *IABP* intra-aortic balloon pump

^a^Continuous variables with normal distribution are presented as mean ± SD unless noted. ^b^Continuous variables of non-normal distribution are presented as median (*P*_25_, *P*_75_). ^c^*P*  < 0.05 was considered as statistically significant

### Logistic regression analysis for risk factors of hyperbilirubinemia after OPCAB

All the potential risk factors were involved in the univariate logistic regression analyses. Based on univariate regression analysis, male (*P* = 0.012), preoperative total bilirubin levels (*P* < 0.001), perioperative IABP implantation (*P*  = 0.037) and perioperative blood transfusion (*P* = 0.008) were risk factors for developing hyperbilirubinemia. Then, multivariate binary logistic regression analysis was performed with variables that had been significant in previous univariate analysis (male, preoperative total bilirubin levels, perioperative IABP implantation and perioperative blood transfusion). Finally, we revealed that elevated preoperative serum total bilirubin levels (OR = 1.225, 95% *CI* 1.145–1.310, *P* < 0.001), perioperative blood transfusion (OR = 4.488, 95% *CI* 1.876–10.737, *P* = 0.001) and perioperative IABP implantation (OR = 4.016, 95% *CI* 1.709–9.439, *P* = 0.001) were independent risk factors for developing hyperbilirubinemia after OPCAB (Table 2).

**Table 2:** Multivariate logistic regression analysis on risk factors of hyperbilirubinemia after OPCAB

Variables	β	OR	95% *CI*	*P*
Preoperative serum total bilirubin (μmol/L)	0.203	1.225	1.145–1.310	< 0.001^a^
Perioperative blood transfusion	1.501	4.488	1.876–10.737	0.001^a^
Perioperative IABP implantation	1.390	4.016	1.709–9.439	0.001^a^

*β* regression coefficient, *OR* odds ratio, *CI* confident interval

^a^*P* < 0.05 was considered as statistically significant

## Discussion

The incidence of hyperbilirubinemia after OPCAB was 7.7%, and the incidence of severe hyperbilirubinemia was 1.4% in this single-center study, which were less than those reported in previous studies, ranging from 13 to 51% [2–7, 9]. The potential reason may be that only patients undergoing OPCAB were enrolled. Since CPB is non-physiological, it will bring a series of stress responses and inflammatory reactions to the body, which may aggravate liver injury and hepatocyte destruction, leading to hepatocellular hyperbilirubinemia. At the same time, CPB can cause inevitable mechanical damage to red blood cells, resulting in hemolytic hyperbilirubinemia [3, 4, 8, 9]. After excluding these potential influence factors, the incidence of hyperbilirubinemia after OPCAB has decreased. This was consistent with our theoretical expectations.

Multivariate logistic regression analysis demonstrated that elevated preoperative serum total bilirubin levels (OR = 1.225, 95% *CI* 1.145–1.310, *P* < 0.001), perioperative blood transfusion (OR = 4.488, 95% *CI* 1.876–10.737, *P* = 0.001) and perioperative IABP implantation (OR = 4.016, 95% *CI* 1.709–9.439, *P* = 0.001) were independent risk factors for developing hyperbilirubinemia after OPCAB. Mild elevation of preoperative bilirubin (exceeding the upper limit of the reference value, but not exceeding twofold) is an independent risk factor for hyperbilirubinemia after OPCAB. Even if the preoperative bilirubin level does not meet the diagnostic criteria of hyperbilirubinemia, and is not accompanied by other manifestations of abnormal liver function such as elevated liver enzymes, decreased albumin or abnormal coagulation function, it is also closely related to postoperative hyperbilirubinemia. The increase of basic serum bilirubin indicates the insufficient reserve of liver function, poorer compensatory ability, and there might be potential hepatocyte injury before surgery. These patients are more likely to have liver dysfunction after surgery, which may cause hepatocyte injury and intrahepatic cholestasis and result in hyperbilirubinemia.

IABP implantation ensures blood perfusion to the heart and brain [15, 16]. On the other hand, it would reduce the perfusion of liver and other organs, resulting in insufficient liver perfusion, ischemia and hypoxia, atrophy and apoptosis of liver cells, which lead to varying degrees of liver dysfunction [2, 17]. Moreover, all stages of the bilirubin circulatory pathway, including albumin transport, hepatocyte uptake, carrier protein transport and related enzyme catalysis, are blocked by inhibition of hepatocyte function, resulting in continuous accumulation of bilirubin in serum [8, 18]. Meanwhile, functions of Kupffer cells of macrophages in hepatic sinuses are inhibited, while their roles in clearing aging, damaged red blood cells, as well as clearing microorganisms and poisons from the intestinal tract are weakened, resulting in pathophysiological changes such as bilirubin stasis and intestinal endotoxemia [19, 20]. The former aggravates inhibition of the roles of Kupffer cells, while the latter can damage the intestinal mucosal barrier through a series of actions and promote endotoxin absorption, thereby leading to a vicious cycle. Endotoxin accumulation may cause hepatocyte injury and induce apoptosis through immune cell activation, induction of the production of free oxygen radicals, secretion of cytokines and inflammatory mediators, inhibiting bilirubin circulation, and finally, by leading to the formation of hepatocyte hyperbilirubinemia [8, 18–20].

Lockey et al. and Wang et al. reported that elevated indirect bilirubin levels were the main causes of postoperative hyperbilirubinemia [7, 17], implied that hemolysis is an important factor in hyperbilirubinemia occurrence and development. Perioperative blood transfusion is a main reason of hemolysis, hemolysis of stored red blood cells can lead to massive accumulation of bilirubin, resulting in postoperative hyperbilirubinemia [21]. Mathie et al. [8] reported that massive blood transfusion may cause microthrombosis, elevated free radical levels and disorders of liver blood flow, further aggravating liver injury and forming hyperbilirubinemia. Moreover, An et al. [9] proposed that prolonged operation time is an independent risk factor for postoperative hyperbilirubinemia, indicating that elevated tissue oozing and bleeding associated with prolonged operation time, intraoperative destruction and loss of red blood cells and coagulation factors may be the potential mechanisms [5].

Regarding the categories of postoperative hyperbilirubinemia, most patients (71.88%) exhibited elevated direct bilirubin levels, which is consistent with previous reports [2, 11]. Nishi et al. reported that patients with elevated direct bilirubin levels were more likely to have higher mortality rates and worse prognostic outcomes than patients with elevated indirect bilirubin levels [11]. This difference was also found in this study (17.39% for direct bilirubin vs. 0% for indirect bilirubin, *P* = 0.303), although the difference was not significant. In contrast, Mastoraki et al. [2] reported that patients with elevated indirect bilirubin levels had worse outcomes. In the hyperbilirubinemia patients of this study, 19 patients (59.38%) had direct bilirubin/total bilirubin ratio > 50%, 10 patients (31.25%) had direct bilirubin/total bilirubin ratio between 20 and 50%, 3 patients (9.38%) had direct bilirubin/total bilirubin ratio < 20%, while 5 patients (15.63%) had elevated liver enzyme levels, demonstrated that the main cause of hyperbilirubinemia after OPCAB was intrahepatic cholestasis, followed by hepatocyte damage, and hemolysis in a few patients. Which is in agreement to the conclusion of Mastoraki et al. [2].

Most studies were positive about the correlation between hyperbilirubinemia and postoperative complications as well as with adverse events [4, 6, 11, 17, 18, 22–24], which embodied the importance of this study. Hyperbilirubinemia after cardiac surgery (especially after cardiopulmonary bypass) is a risk factor for postoperative respiratory [9], circulatory [18, 24], urinary [25, 26], gastrointestinal complications [27] and associated with in-hospital mortality [2–7, 9].

The mechanisms through which hyperbilirubinemia leads to complications and poor prognosis are complex, and have not been fully elucidated. Elevated bilirubin concentrations can trigger cell oxidative stress, induce apoptosis [23], and participate in a variety of pathological processes, including respiratory dysfunction, thrombocytopenia [25], renal tubular epithelial cell damage [26, 28], neural cell differentiation and myelination inhibition [29]. In addition, persistent hyperbilirubinemia is only a manifestation of a potential disease, such as persistent low cardiac output syndrome, rather than a direct cause of death [3].

This study has certain significance for clinical practice. Firstly, for patients with elevated serum total bilirubin levels before OPCAB, potential causes should be investigated and early therapy for the primary disease should be administered. Secondly, the experience level of the surgical team should be improved to reduce iatrogenic complications of IABP implantation, decrease intraoperative bleeding, shorten operation time, so as to suppress the chances of perioperative hyperbilirubinemia caused by iatrogenic factors. Thirdly, there is a need to improve the management ability of blood products. For patients with high risk of bleeding and transfusion, autologous blood transfusion and a restrictive transfusion strategy [21, 30] should be adopted. Moreover, regular measurement of postoperative serum total bilirubin levels is necessary. For patients with mild elevations of serum total bilirubin, choleretic drugs such as ursodeoxycholic acid and transmetil could be effective. Meanwhile, hepatoprotective medicine such as glutathione, polyene phosphatidylcholine, magnesium isoglycyrrhizinate would be administrated. For patients with severe elevations of serum total bilirubin and acute liver failure, artificial liver support system (ALSS), including plasma bilirubin adsorption (PBA), plasma exchange (PE), hemofiltration (HF), hemodialysis (HD) and their combination strategies should be applied, according to the indications. If the progress of hyperbilirubinemia cannot be controlled by the above methods, liver transplantation may be the last chance [31, 32].

This study had some limitations. First, as a single center retrospective study, the sample size was still limited. Then, we did not record factors such as the volume and component of transfusion, time of IABP use, which may affect the results of this study. Consequently, further studies are need to verify the risk factors of developing hyperbilirubinemia after OPCAB.

## Conclusion

In this study, we revealed that hyperbilirubinemia was also a common complication after OPCAB. Elevated preoperative serum total bilirubin levels, perioperative IABP implantation and perioperative blood transfusion were independent risk factors for hyperbilirubinemia after OPCAB. In clinical practice, these factors should be significantly focused and prevented to reduce the occurrence of postoperative hyperbilirubinemia after OPCAB.

## Data Availability

The datasets used or analyzed during the current study are available from the corresponding author on reasonable request.

## Abbreviations

ALP: Alkaline phosphatase
ALSS: Artificial liver support system
ALT: Alanine aminotransferase
AST: Aspartate aminotransferase
BMI: Body mass index
CABG: Coronary artery bypass grafting
*CI*: Confidence interval
COPD: Chronic obstructive pulmonary disease
CPB: Cardiopulmonary bypass
GGT: γ-Glutamyl transpeptidase
HD: Hemodialysis
HF: Hemofiltration
IABP: Intra-aortic balloon pump
LAD: Left anterior descending artery
LAD: Left atrial diameter
LIMA: Left internal mammary artery
LVEDD: Left ventricular end diastolic diameter
LVEF: Left ventricle ejection fraction
OPCAB: Off-pump coronary artery bypass grafting
OR: Odds ratio
PBA: Including plasma bilirubin adsorption
PE: Plasma exchange

## References

[1] Altarabsheh SE, Deo SV, Rababa'H AM, Lim JY, Cho YH, Sharma V (2015). Off-pump coronary artery bypass reduces early stroke in octogenarians: a meta-analysis of 18,000 patients. Ann Thorac Surg.

[2] Mastoraki A, Karatzis E, Mastoraki S, Kriaras I, Sfirakis P, Geroulanos S (2007). Postoperative jaundice after cardiac surgery. Hepatobiliary Pancreat Dis Int HBPD INT.

[3] Sharma P, Ananthanarayanan C, Vaidhya N, Malhotra A, Shah K, Sharma R (2015). Hyperbilirubinemia after cardiac surgery: an observational study. Asian Cardiovasc Thorac Ann.

[4] Collins JD, Bassendine MF, Ferner R, Blesovsky A, Murray A, Pearson DT (1983). Incidence and prognostic importance of jaundice after cardiopulmonary bypass surgery. Lancet (London, England).

[5] Hosotsubo KK, Nishimura M, Nishimura S (2000). Hyperbilirubinaemia after major thoracic surgery: comparison between open-heart surgery and oesophagectomy. Crit Care (London, England).

[6] Chu CM, Chang CH, Liaw YF, Hsieh MJ (1984). Jaundice after open heart surgery: a prospective study. Thorax.

[7] Lockey E, McIntyre N, Ross DN, Brookes E, Sturridge MF (1967). Early jaundice after open-heart surgery. Thorax.

[8] Mathie RT (1993). Hepatic blood flow during cardiopulmonary bypass. Crit Care Med.

[9] An Y, Xiao YB, Zhong QJ (2006). Hyperbilirubinemia after extracorporeal circulation surgery: a recent and prospective study. World J Gastroenterol.

[10] Chen X, Bai M, Zhao L, Li Y, Yu Y, Zhang W (2020). Characteristics and outcomes of Stanford type A aortic dissection patients with severe post-operation hyperbilirubinemia: a retrospective cohort study. J Cardiothorac Surg.

[11] Nishi H, Sakaguchi T, Miyagawa S, Yoshikawa Y, Fukushima S, Saito S (2012). Frequency, risk factors and prognosis of postoperative hyperbilirubinemia after heart valve surgery. Cardiology.

[12] Dehmer GJ, Badhwar V, Bermudez EA, Cleveland JC, Cohen MG, D Agostino RS, et al. 2020 AHA/ACC Key Data Elements and Definitions for Coronary Revascularization: A Report of the American College of Cardiology/American Heart Association Task Force on Clinical Data Standards (Writing Committee to Develop Clinical Data Standards for Coronary Revascularization). Circ Cardiovasc Qual Outcomes 2020;13(4):e000059. 10.1161/HCQ.0000000000000059 32202924

[13] Sullivan JI, Rockey DC (2017). Diagnosis and evaluation of hyperbilirubinemia. Curr Opin Gastroenterol.

[14] Haematology TL (2016). Updates on blood transfusion guidelines. Lancet Haematol.

[15] Huu AL, Shum-Tim D (2018). Intra-aortic balloon pump: current evidence & future perspectives. Future Cardiol.

[16] Stone GW, Ohman EM, Miller MF, Joseph DL, Christenson JT, Cohen M (2003). Contemporary utilization and outcomes of intra-aortic balloon counterpulsation in acute myocardial infarction. J Am Coll Cardiol.

[17] Wang MJ, Chao A, Huang CH, Tsai CH, Lin FY, Wang SS (1994). Hyperbilirubinemia after cardiac operation. Incidence, risk factors, and clinical significance. J Thorac Cardiovasc Surg.

[18] Allen LA, Felker GM, Pocock S, McMurray JJ, Pfeffer MA, Swedberg K (2009). Liver function abnormalities and outcome in patients with chronic heart failure: data from the Candesartan in Heart Failure: Assessment of Reduction in Mortality and Morbidity (CHARM) program. Eur J Heart Fail.

[19] Lin HC, Yang YY, Tsai TH, Huang CM, Huang YT, Lee FY (2011). The relationship between endotoxemia and hepatic endocannabinoids in cirrhotic rats with portal hypertension. J Hepatol.

[20] Lough J, Rosenthall L, Arzoumanian A, Goresky CA (1987). Kupffer cell depletion associated with capillarization of liver sinusoids in carbon tetrachloride-induced rat liver cirrhosis. J Hepatol.

[21] Chen QH, Wang HL, Liu L, Shao J, Yu J, Zheng RQ (2018). Effects of restrictive red blood cell transfusion on the prognoses of adult patients undergoing cardiac surgery: a meta-analysis of randomized controlled trials. Crit Care (London, England).

[22] Farag M, Veres G, Szabó G, Ruhparwar A, Karck M, Arif R (2019). Hyperbilirubinaemia after cardiac surgery: the point of no return. ESC Heart Fail.

[23] Chintanaboina J, Haner MS, Sethi A, Patel N, Tanyous W, Lalos A (2013). Serum bilirubin as a prognostic marker in patients with acute decompensated heart failure. Korean J Intern Med.

[24] Shinagawa H, Inomata T, Koitabashi T, Nakano H, Takeuchi I, Naruke T (2008). Prognostic significance of increased serum bilirubin levels coincident with cardiac decompensation in chronic heart failure. Circ J.

[25] NaveenKumar SK, Thushara RM, Sundaram MS, Hemshekhar M, Paul M, Thirunavukkarasu C (2015). Unconjugated bilirubin exerts pro-apoptotic effect on platelets via p38-MAPK activation. Sci Rep.

[26] Chen X, Bai M, Zhao L, Yu Y, Yue Y, Sun S (2021). Time to peak bilirubin concentration and advanced AKI were associated with increased mortality in rheumatic heart valve replacement surgery patients with severe postoperative hyperbilirubinemia: a retrospective cohort study. BMC Cardiovasc Disord.

[27] McSweeney ME, Garwood S, Levin J, Marino MR, Wang SX, Kardatzke D (2004). Adverse gastrointestinal complications after cardiopulmonary bypass: can outcome be predicted from preoperative risk factors?. Anesth Analg.

[28] Yuan L, Liao PP, Song HC, Zhou JH, Chu HC, Lyu L (2019). Hyperbilirubinemia induces pro-apoptotic effects and aggravates renal ischemia reperfusion injury. Nephron.

[29] Barateiro A, Domingues HS, Fernandes A, Relvas JB, Brites D (2014). Rat cerebellar slice cultures exposed to bilirubin evidence reactive gliosis, excitotoxicity and impaired myelinogenesis that is prevented by AMPA and TNF-α inhibitors. Mol Neurobiol.

[30] Akhlagh SH, Vaziri MTM, Nemati MH, Ashraf H (2011). Changes in liver enzymes and bilirubin after coronary artery bypass grafting using acute normovolemic hemodilution. Acta Anaesthesiol Belg.

[31] Wendon J, Cordoba J, Dhawan A, Larsen FS, Manns M, Samuel D (2017). EASL Clinical Practical Guidelines on the management of acute (fulminant) liver failure. J Hepatol.

[32] Flamm SL, Yang YX, Singh S, Falck-Ytter YT (2017). American gastroenterological association institute guidelines for the diagnosis and management of acute liver failure. Gastroenterology.

